# Correction: An exchange coupled *meso*–*meso* linked vanadyl porphyrin dimer for quantum information processing

**DOI:** 10.1039/d2sc90246j

**Published:** 2022-11-30

**Authors:** Davide Ranieri, Fabio Santanni, Alberto Privitera, Andrea Albino, Enrico Salvadori, Mario Chiesa, Federico Totti, Lorenzo Sorace, Roberta Sessoli

**Affiliations:** Department of Chemistry “Ugo Schiff” & INSTM RU, University of Florence Via della Lastruccia 3 50019 Sesto Fiorentino Italy lorenzo.sorace@unifi.it; Department of Chemistry, NIS, University of Turin Via P. Giuria 7 I10125 Torino Italy

## Abstract

Correction for ‘An exchange coupled *meso*–*meso* linked vanadyl porphyrin dimer for quantum information processing’ by Davide Ranieri *et al.*, *Chem. Sci.*, 2022, https://doi.org/10.1039/d2sc04969d.

During the editorial production of the finished article, the graphics associated with Fig. 3(a) and (b) were inadvertently switched.

The intended version of Fig. 3 is shown below, and replaces that of the original article:

**Fig. 3:**
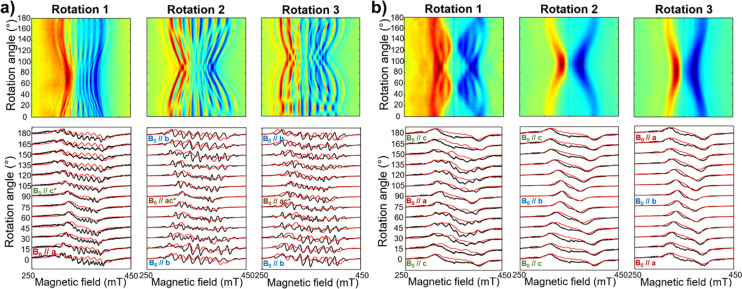
Room temperature angular-dependent CW EPR X-band spectra of (a) ***m*-[VO(TrPP)]_2_** and (b) ***o*-[VO(TrPP)]_2_** for crystal rotations around three orthogonal axes. For both panels, the upper row shows the 2D experimental EPR contour plots for the three rotations, acquired with a 3° step; the lower row shows representative EPR spectra (black lines) for the three rotations – from 0° to 180° every 15° – together with the best spectral simulations (red lines) obtained by using |*J*| = 0.01 (0.005) cm^−1^ and |*J*| = 0.05 (0.01) cm^−1^ for (a) and (b), respectively. Experimental frequency: 9.40 GHz for (a), 9.87 GHz for (b).

The Royal Society of Chemistry apologises for these errors and any consequent inconvenience to authors and readers.

## Supplementary Material

